# Broadband Spin‐Locked Metasurface Retroreflector

**DOI:** 10.1002/advs.202201397

**Published:** 2022-05-11

**Authors:** Qingze Tan, Bin Zheng, Tong Cai, Chao Qian, Rongrong Zhu, Xiaofeng Li, Hongsheng Chen

**Affiliations:** ^1^ Interdisciplinary Center for Quantum Information State Key Laboratory of Modern Optical Instrumentation ZJU‐Hangzhou Global Scientific and Technological Innovation Center Zhejiang University Hangzhou 310027 China; ^2^ International Joint Innovation Center Key Lab. of Advanced Micro/Nano Electronic Devices & Smart Systems of Zhejiang The Electromagnetics Academy at Zhejiang University Zhejiang University Haining 314400 China; ^3^ School of Information and Electrical Engineering Zhejiang University City College Hangzhou 310015 China; ^4^ Jinhua Institute of Zhejiang University Zhejiang University Jinhua 321099 China; ^5^ Air and Missile Defend College Air force Engineering University Xi'an 710051 China

**Keywords:** broadband spectrum, retroreflection, single‐layer metasurface, spin‐locked retroreflector

## Abstract

Retroreflectors are ubiquitously used in a multitude of applications, such as cloaking, wireless communication, radar, and antenna, owing to their ability to augment the reflected electromagnetic (EM) waves in the incident direction. However, Current metasurface retroreflector designs have yet to mature into a practical method due to the limitations of low efficiency and narrow band, which actually originate from the difficulty in simultaneously engineering phase profiles of certain metasurface at distinct wavelengths. Here, a broadband spin‐locked retroreflector with high efficiency that relies only on a simple metasurface layer is demonstrated. The metasurface is designed with low‐loss dielectric resonators, introducing both the propagation and geometric phases to enable dispersive phase compensation. The results indicate that the proposed metasurface can achieve retroreflection over a broadband spectrum while keeping the spin state identical. Furthermore, a broadband spin‐locked cloak is presented for validation. The work builds up a major advance for practice‐oriented retroreflector and even envision this approach may open new vistas in the very cutting‐edge research of 6G wireless communication network.

## Graphical abstract

A broadband spin‐locked retroreflector is demonstrated with high efficiency that relies only on a single‐layer metasurface. The metasurface is designed with low‐loss dielectric resonators, introducing both the propagation and geometric phases to enable dispersive phase compensation.

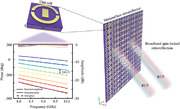

## 1 Introduction

Retroreflector is an important electromagnetic (EM) device or surface that can reflect incident EM waves back as it comes. It has attracted significant research attention owing to its advantages in communication systems, remote sensing technology, directional energy transmission and so on. Conventional retroreflectors are typically designed using corner reflectors^[^ ^1^, ^2^ ^]^ or Luneburg lenses,^[^ ^3^, ^4^ ^]^ which are generally bulky and nonplanar; therefore, they are not suitable for miniaturization and integration. With the development of metamaterial,^[^ ^5^, ^6^, ^7^, ^8^, ^9^, ^10^, ^11^, ^12^, ^13^ ^]^ arbitrary controlling propagation of waves has been possible. In recent years, metasurfaces have emerged as versatile platforms owing to their excellent ability to manipulate light.^[^ ^14^, ^15^, ^16^, ^17^ ^]^ Because the phase of the metasurface can be accurately controlled by subwavelength‐scale structures, many optical functional devices and applications, including metalenses,^[^ ^18^, ^19^, ^20^, ^21^, ^22^ ^]^ polarization convertors,^[^ ^23^, ^24^, ^25^, ^26^ ^]^ holograms,^[^ ^27^, ^28^, ^29^ ^]^ and cloaking,^[^ ^30^, ^31^, ^32^, ^33^, ^34^, ^35^, ^36^ ^]^ have been demonstrated. Moreover, the advent of metasurfaces has made feasible the realization of extremely thin retroreflectors.^[^ ^37^, ^38^, ^39^, ^40^, ^41^, ^42^ ^]^ Furthermore, metasurfaces with spin‐based applications^[^ ^43^, ^44^, ^45^, ^46^, ^47^, ^48^, ^49^, ^50^, ^51^, ^52^, ^53^ ^]^ have attracted significant interest owing to the cutting‐edge research field of spin optics.^[^ ^54^, ^55^ ^]^ The spin of light refers to the right‐ and left‐handed circular polarization (RCP and LCP) of a light beam, where the handedness depends on the direction of propagation. Spin‐locked retroreflectors based on metasurfaces have been reported,^[^ ^56^, ^57^, ^58^ ^]^ but they are limited to a single wavelength or very narrow spectrum. Additionally, experimental work on spin‐locked metasurface retroreflectors in broadband spectrum is still elusive.

Here, we demonstrate a broadband spin‐locked retroreflector with high efficiency, which employs the low‐loss resonant unit element in metasurface with smooth and linear phase dispersion. This is made possible by combining the propagation and geometric phases to implement dispersive phase compensation. The proposed retroreflector maintains the handedness of the reflected waves, which is the same as the incidence. The simulated and experimental results prove the feasibility of the broadband spin‐locked retroreflector concept. Moreover, a spin‐locked cloak based on the designed retroreflector is demonstrated to restore completely the distorted phase of EM waves caused by bumps in the broadband spectrum. The proposed retroreflector has great practical potential in wireless communication systems that utilize spin‐based retroreflection devices.

## 2 Results

According to the generalized laws of reflection,^[^ ^59^ ^]^ for retroreflection, *θ*_*i*_ = −*θ*_*r*_, where *θ*_*i*_ is the incident angle and *θ*_*r*_ is the angle of reflection. We can obtain the angle of reflection as

(1) $$\theta_{r}=\sin^{-1}\left(\frac{\lambda }{4\pi }\frac{d\phi }{dx}\right)=\sin^{-1}\left(\frac{c}{4\pi f}\frac{\Delta \varphi (f)}{p}\right)$$

where *λ* is the wavelength of the EM wave, $\frac{d\phi }{dx}$ is a suitable phase gradient, *c* is the velocity of light in vacuum, *f* is the working frequency, Δ*ϕ*(*f*) is the phase shift between two adjacent unit cells along the interface, and *p* is the period of the unit cell. To realize broadband retroreflection, Equation (2) should be satisfied.

(2) $$\frac{\Delta \varphi (f)}{f}=\mathrm{constant}$$

**Figure 1:**
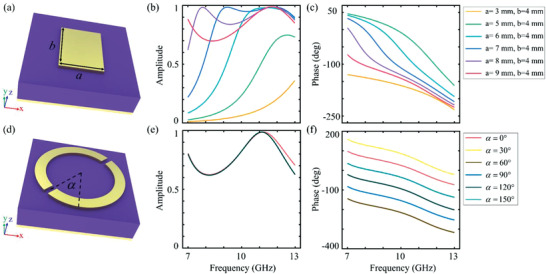
a) Schematic of the RPR unit. a and b represent the width and length of the top patch. b,c) Amplitude and phase responses of (a). Different color lines represent different values of a and b (the specific values are listed in Table S1, Supporting Information). d) Schematic of the split ring resonator unit, where *α* represents the rotation angle of top split ring. e,f) Amplitude and phase responses of (d). Different color lines represent different values of *α* (the specific values are listed in Table S2 in the Supporting Information). All simulated results are obtained under the condition that incidence and reflection are both right‐handed circular polarized waves.

To solve the crucial issue of the phase profiles required in Equation (2), we design a spin‐locked single‐layer metasurface composed by exquisitely constructed unit cells. The unit cell is composed of a composite split ring resonator (SRR) and a rectangular patch resonator (RPR), as shown in **Figures** **1**a–d, respectively. The SRR and RPR are on a substrate of thickness 2 mm (0.06*λ*) and relative permittivity of 4.3 + 0.004i. The backside of the unit cell is a metallic plane. The period, *p*, is set to 10 mm. This single‐layer metasurface can accurately meet the requirement of desired phase distribution by simultaneously adjusting propagation and geometric phases.^[^ ^60^, ^61^ ^]^ For the propagation phase alone, the reflected phase gradients can be changed by adjusting the structural parameters (a and b, refer to Supporting Information) of the RPR, where Figure 1b,c are the amplitude and phase response with respect to the situation in which incidence and reflection are both RCP waves. Moreover, for the geometric phase, the phase response can be changed by rotating the SRR (*α*, refer to Supporting Information) at fixed structural parameters. As can be seen in Figure 1e,f, the phase gradients remain almost the same when the phase profile shifts. According to the characteristics of propagation and geometric phases, we employ a unit cell that combines both SRR and RPR structures to obtain the phase distribution that satisfies the requirement of the broadband retroreflector and simultaneously ensures high efficiency of the spin‐locked feature as well.

**Figure 2:**
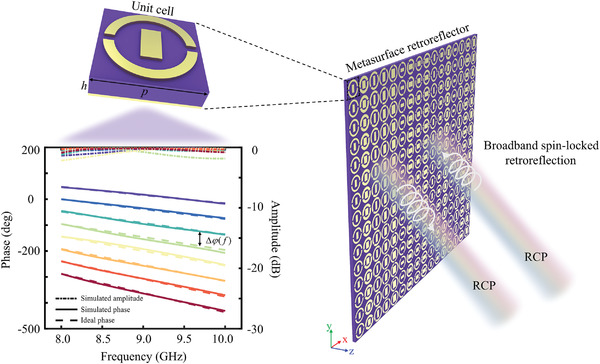
Design and schematic representation of the broadband spin‐locked metasurface retroreflector. When the broadband RCP waves impinge on the metasurface, it will be reflected along the incident direction with the same spin state. The top inset is the unit cell, where *p* = 10 mm and *h* = 2 mm. The bottom inset shows reflected amplitude (dotted line) and phase response (solid line) of the eight unit cells, which compose the entire metasurface. The different colors represent the eight different unit cells. Δ*ϕ*(*f*) is the value of phase discontinuity between two adjacent unit cells along the interface.

A schematic of the designed unit cell is shown in the inset of **Figure** **2**. We use eight unit cells with a constant phase gradient (detailed structural parameters are listed in Table S3,Supporting Information). Both incidence and reflection are RCP waves, and the amplitude and phase responses of the designed eight unit cells are shown in the bottom inset of Figure 2. The solid lines represent the simulated phase, which coincides well with the dotted lines that satisfy $\frac{\Delta \varphi (f)}{f}=6$ (°/GHz). Moreover, the conversion efficiency is >80%, which guarantees the spin‐locked characteristic. A schematic of the proposed broadband spin‐locked retroreflector is shown in Figure 2. This retroreflector can reflect the EM waves along its incident direction in a broadband frequency spectrum; moreover, it can maintain an identical spin state.

**Figure 3:**
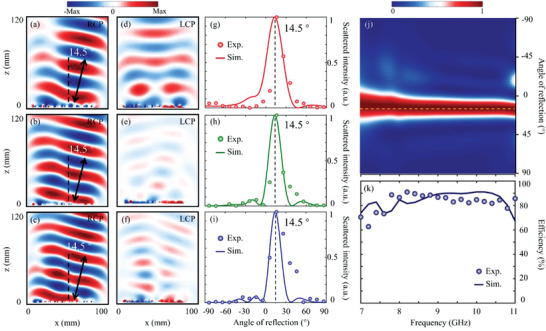
a–c) Simulated electric field distributions of reflected RCP waves under RCP illumination with an incident angle of 14.5° at a) 8 GHz, b) 9 GHz, and c) 10 GHz. d–f) Simulated electric field distributions of reflected LCP waves under RCP illumination with an incident angle of 14.5° at a) 8 GHz, b) 9 GHz, and c) 10 GHz. g–i) Measured (symbols) and simulated (solid line) normalized scattering intensity versus the reflection angle under RCP waves illumination with an incident angle of 14.5° at g) 8, h) 9, and i) 10 GHz. Different colors represent different working frequencies. j) Simulated scattered‐field intensity (color map) versus frequency and angles under RCP incidence with an incident angle of 14.5°. k) Simulated (solid line) and measured (symbols) absolute efficiencies of the designed retroreflector.

The metasurface is feasible for realizing retroreflection of the broadband spectrum while locking the spin characteristics of the EM waves. Based on Equation (2), a spin‐locked retroreflector with 14.5° is realized in the range 8–10 GHz using the designed metasurface, and simulation is performed in CST Microwave Studio. **Figure** **3**a–c illustrates the electric field distributions of the reflected RCP waves in the *xoz* plane at 8, 9, and 10 GHz. As expected, the very clean beam‐bending signature and the exact deflection angle of 14.5° reinforce our notion of achromatism over a wide bandwidth. Figure 3d–f illustrates the electric field distributions of the reflected LCP waves in the *xoz* plane under RCP incidence with an incident angle of 14.5°, which demonstrates the spin‐locked feature of the designed retroreflector. Furthermore, the far‐field patterns of the spin‐locked broadband retroreflector are experimentally investigated. The SRR and RPR are printed on a 2 mm epoxy glass cloth copper‐clad laminate with a permittivity of 4.3, there are 30 unit cells along the y direction. The far‐field experiment is carried out in an anechoic chamber. RCP waves from the circularly polarized horn antenna are obliquely incident on the retroreflector with the incident angle of 14.5°. Another circularly polarized horn antenna works as the receiver to measure the scattered fields with a distance of 2.0 m. All the scattered intensities are normalized to their own maxima to improve the presentation of the scattering direction. Figures 3g–i show the simulated and experimental results of the normalized far‐field scattered intensity under RCP wave incidence with an incident angle of 14.5°. The measured results coincide well with the simulated results, with the maximum scattered intensity at different measured frequencies appearing at 14.5°. Therefore, all the incident power is transferred to the desired direction under RCP illumination, which is highly consistent with the theoretical prediction. The scattering patterns are simulated within the operating frequency band as well. As shown in Figure 3j, within the target frequency band, all the incident RCP waves are reflected to an identical angle of 14.5° as predesigned. Figure 3k shows the experimental and simulated efficiencies of the retroreflector as a function of frequency. The efficiency of the retroreflector is the ratio between the power of the desired reflected direction and the incident power at different frequencies.^[^ ^62^ ^]^ At the target frequency band, the absolute efficiencies of the designed retroreflector range from 81% to 91%, as retrieved from our experimental results. The slight difference between the simulation and experimental results may be attributed to inevitable fabrication errors. All of the aforementioned results verify the feasibility of the designed spin‐locked retroreflector for broadband spectra.

**Figure 4:**
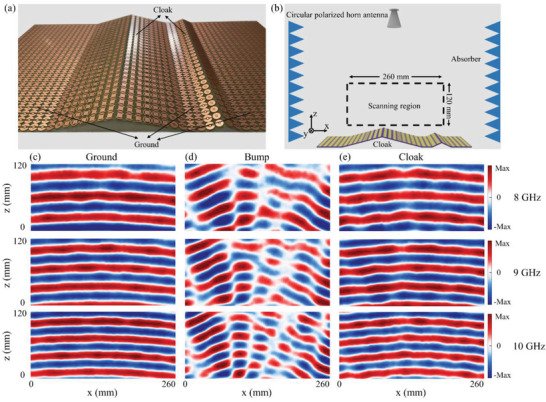
a) Experimental sample of the metasurface cloak. b) Experimental setup. RCP waves from the circular polarized horn antenna are normally incident on the cloaked bump. The measured region (260 mm  ×  120 mm) is in the *x*o*z* plane at *y* = 0 (the cloak is symmetric at *y* = 0) over the cloaked bump. c) Measured reflected electric field distributions of RCP waves of ground at 8, 9, 10 GHz. d) Measured reflected electric field distributions of RCP waves of bare bump at 8, 9, 10 GHz. e) Measured reflected electric field distributions of RCP waves of cloaked bump at 8, 9, 10 GHz.

Moreover, we build a broadband spin‐locked metasurface cloak using the designed retroreflector. **Figure** **4**a shows a photograph of the fabricated cloak. It is composed of two types of retroreflectors with the inclinations of 14.5° and 24.6°. In particular, the smaller retroreflector with an inclination of 24.6° has two unit cells (the detailed structural parameters are listed in Table S3, Supporting Information). The remaining flat parts are spin‐locked ground composed of identical unit elements to maintain the spin state. In the experimental setup, RCP waves from the circularly polarized horn antenna are normally incident on the metasurface cloak sample, as shown in Figure 4b. The measurements are performed in an anechoic chamber. The electric field at an area of 260  ×  120 mm in the *xoz* plane (the sample is symmetric ≈*y* = 0) over the samples are measured. Figure 4c,d shows the measured electric field of the reflected RCP waves for flat spin‐locked ground and bare bumps, respectively. For comparison, the bump is composed of the same identical unit cells as the spin‐locked ground to keep the spin characteristics of the EM waves unchanged (as shown in Figures S3 and S4, Supporting Information). It shows that when the EM waves impinge on bare bumps, the reflected beams are distorted and scattered into different directions. After covering the bump with the designed cloak, the distortion of the reflected phase is reconstructed and the split beams rejoin again, as shown in Figure 4e. One can see the measured results of the cloaked bump are similar to those measured from the spin‐locked ground in Figure 4c, indicating that a single layer of the skin cloak reroutes the waves and restores the wavefront scattered from the bump by compensating the phase difference. Therefore, the reflected waves of bump covered by cloak are same as those of ground as if the bump disappears. It should be noted that there exist some small wavefront distortions which are caused by discontinuous compensated phase and fabrication errors. These small distortions can be reduced with smaller period of unit elements and more precise fabrication.

## 3 Conclusion

In summary, we demonstrate a broadband retroreflector made of ultrathin spin‐locked metasurfaces working at 8–10 GHz with high efficiency. The required phase profile of the unit elements for realizing broadband retroreflection is achieved by incorporating the geometric and propagation phases. The simulated and experimental results indicate that the designed retroreflector has a good performance within the broadband spectrum while keeping the spin state identical. Furthermore, we realize a broadband spin‐locked cloak that can restore the distorted phase of light by utilizing the designed retroreflector. Our design paves the way to a practical class of retroreflectors that can control the phase dispersion at will, and the demonstrated broadband spin‐locked retroreflectors are promising in the applications of spin‐based information transmission systems.

## Conflict of Interest

The authors declare no conflict of interest.

## Supporting information

Supporting Information

## Data Availability

The data that support the findings of this study are available from the corresponding author upon reasonable request.
