# Supplementary material for: Broadband Spin‐Locked Metasurface Retroreflector
Source: Adv Sci (Weinh). 2022 May 11;9(20):2201397. doi: 10.1002/advs.202201397 (PMC9284148; doi:10.1002/advs.202201397)
Supplement: Supplementary file 1 — Supporting Information [file ADVS-9-2201397-s001.pdf]

ADVANCED SCIENCE
Open Access

Supporting Information

for *Adv. Sci.*, DOI 10.1002/advs.202201397

Broadband Spin-Locked Metasurface Retroreflector

Qingze Tan, Bin Zheng, Tong Cai*, Chao Qian, Rongrong Zhu, Xiaofeng Li and Hongsheng Chen**

Supplementary Material for

Broadband Spin-Locked Metasurface Retroreflector

Qingze Tan[1,2,4], Bin Zheng[1,2,4 *], Tong Cai[1,5], Chao Qian[1,2,4], Rongrong Zhu[1,3], Xiaofeng Li[1,5], Hongsheng Chen[1,2,4, *]

[1] *Interdisciplinary Center for Quantum Information, State Key Laboratory of Modern Optical Instrumentation, ZJU-Hangzhou Global Scientific and Technological Innovation Center, Zhejiang University, Hangzhou, 310027, China.*

[2] *International Joint Innovation Center, Key Lab. of Advanced Micro/Nano Electronic Devices & Smart Systems of Zhejiang, The Electromagnetics Academy at Zhejiang University, Zhejiang University, Haining, 314400, China.*

[3] *School of Information and Electrical Engineering, Zhejiang University City College, Hangzhou, 310015, China*

[4] *Jinhua Institute of Zhejiang University, Zhejiang University, Jinhua, 321099, China*

[5] *Air and Missile Defend College, Air force Engineering University, Xi'an, 710051, China*

[*] *Correspondence and requests for materials should be addressed to B. Zheng. (email: zhengbin@zju.edu.cn) and H. Chen. (email: hansomchen@zju.edu.cn).*

The derivation process of Eq. (1) in manuscript.

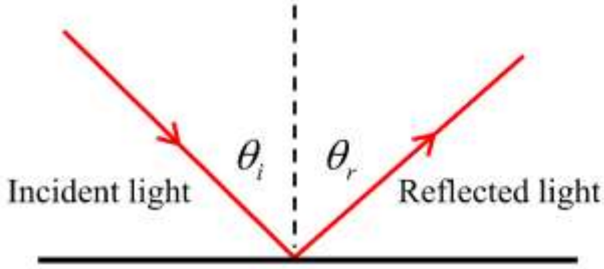

Figure S1. The schematic of light reflection.

For the realization of broadband retroreflector, according to the generalized laws of reflecton

$$\sin(\theta_r) - \sin(\theta_i) = \frac{\lambda}{2\pi n}\frac{d\phi}{dx} \tag{1}$$

where θ_i is the incident angle, θ_r is the angle of reflection, λ is the wavelength of EM wave, n is the refractive indices of media, and in our case $n_{air} = 1$, $\frac{d\phi}{dx}$ is a suitable constant gradient of phase discontinuity along the interface. And for the retroreflection,

$$\theta_i = -\theta_r \tag{2}$$

Then we can get the angle of reflection,

$$\theta_r = \sin^{-1}(\frac{\lambda}{4\pi}\frac{d\phi}{dx}) = \sin^{-1}(\frac{c}{4\pi f}\frac{\Delta\varphi(f)}{p}) \tag{3}$$

Where c is the velocity of light in vacuum, f is working frequency, $\Delta\varphi(f)$ is the phase discontinuity between two adjacent unit cells, and p is the period of unit cell. It is clear from the Eq. (3), for realizing broadband retroreflection, $\frac{\Delta\varphi(f)}{f}$ must be constant.

Structural parameters of RPR and SRR.

The structural parameters of RPR and SRR are listed in Table S1 and Table S2, different colors are corresponding to the lines in Figure 1 (b, c, e, f) in main text.

Table S1. The spatial parameters of RPR shown in Figure 1.

RPR	1	2	3	4	5	6
a (mm)	3	5	6	7	8	9
b (mm)	4	4	4	4	4	4

Table S2. The spatial parameters of SRR shown in Figure 1.

SRR	**1**	**2**	**3**	4	5	6
α (deg)	0	30	60	90	120	150

Structural parameters of unit cells.

Figure S1 shows the specific parameters of unit cell, it is composed of RPR and SRR.

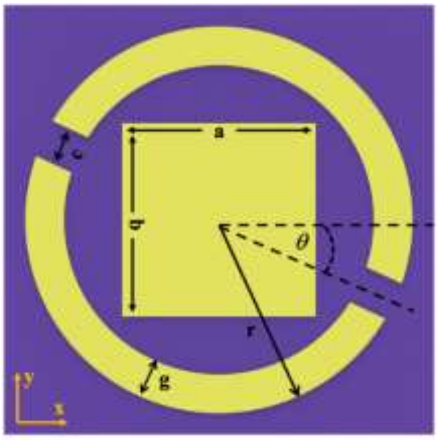

Figure S2. Top view of unit cell which composes the metasurface.

Due to the symmetry, there are 10 different elements in designed cloak as shown in Figure S2, the detailed spatial parameters are listed in Table S3.

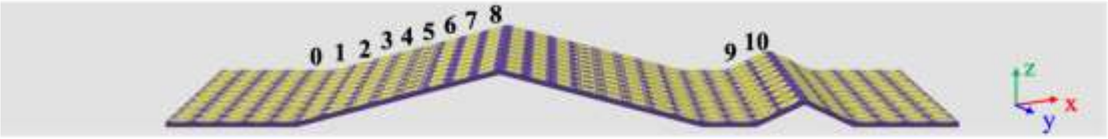

Figure S3. The designed metasurface cloak which includes 10 different unit cells.

Table S3. The spatial parameters of 10 unit cells shown in Figure S2.

Unit cell	0	1	2	3	4	5	6	7	8	9	10
r (mm)	9.9	9.9	9.0	9.9	9.9	9.9	9.9	9.9	9.9	9.8	8.5
g (mm)	3	3	3	0.9	0.9	0.9	0.9	0.9	0.9	3	3
c (mm)	2.3	1.8	0.3	0.9	0.2	3.7	5.9	5.5	5.8	1.0	0.1
a (mm)	2	2	1.5	5.5	5.2	5.5	5.6	5.5	5.6	2.5	1.5
b (mm)	2.5	2.5	1.5	5.5	5.4	5.5	5.5	5.5	5.6	2	1.5
θ (deg)	0	7	37	25	47	91	161	174	10	6	72

The simulated result of unit cell under illumination with different working angle.

As an example, we give the simulated results of reflected amplitude and phase response of the unit cell-4 with different incident angle, shown in Figure S4. We can tell that there is little difference among these results under illumination with different working angle. Therefore, there almost has no effect when we simulated unit cells under illumination with normal

incidence.

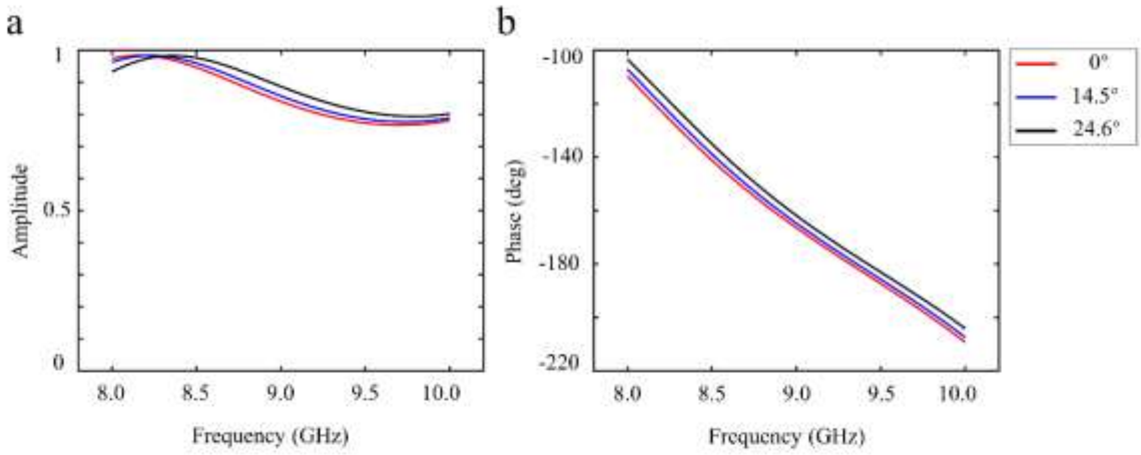

Figure S4. The simulated results of reflected amplitude and phase response of the unit cell-4. The different colors represent different incident angle.

__**The pictures of fabricated ground and bumps.**__

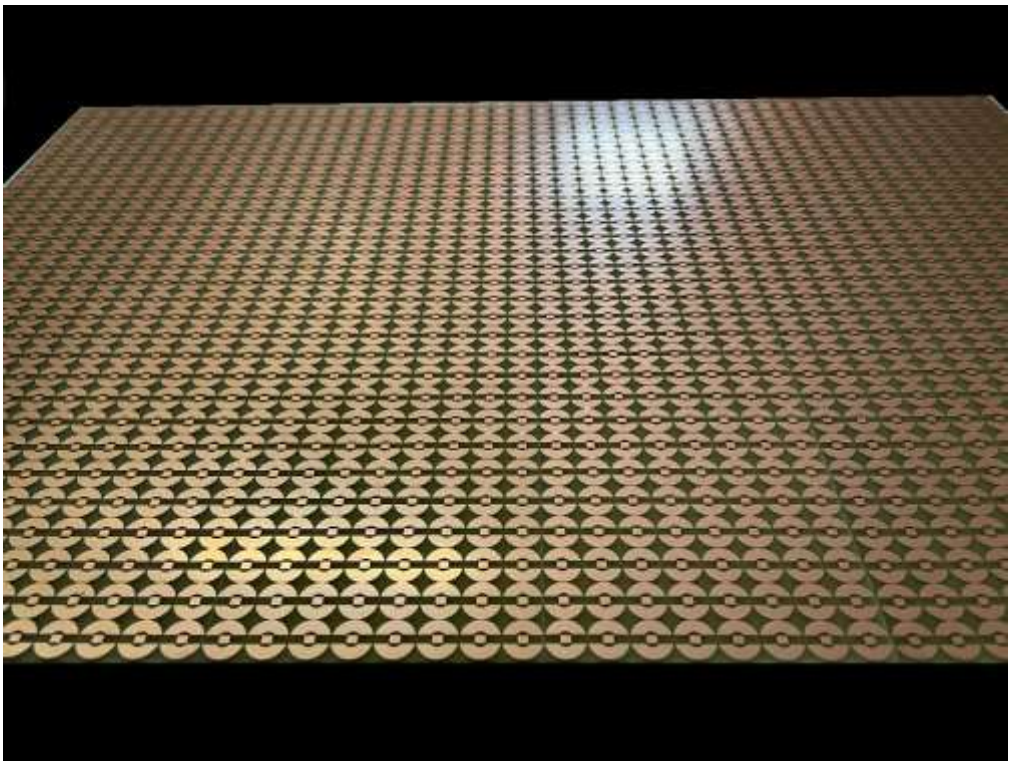

Figure S5. The picture of fabricated spin-locked ground.

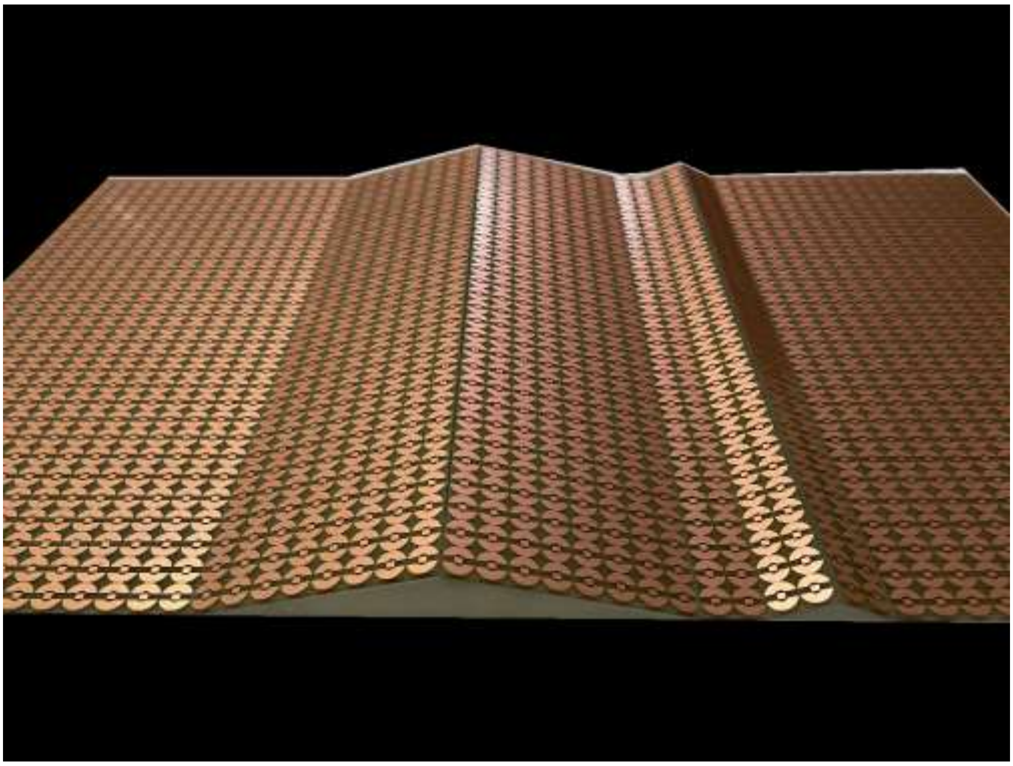

Figure S6. The picture of the fabricated spin-locked bump.
